# GLUT1 as a Potential Therapeutic Target in Glioblastoma

**DOI:** 10.3390/brainsci15060585

**Published:** 2025-05-28

**Authors:** FNU Ruchika, Sanika Suvarnapathaki, Antolin Serrano-Farias, Chetan Bettegowda, Jordina Rincon-Torroella

**Affiliations:** Department of Neurosurgery, School of Medicine, Johns Hopkins University, Baltimore, MD 21287, USA; ruchik1@jh.edu (F.R.); ssuvarn1@jh.edu (S.S.); aserran7@jh.edu (A.S.-F.); cbetteg1@jhmi.edu (C.B.)

**Keywords:** GLUT1, Warburg effect, glioblastoma, hypoxia

## Abstract

Glioblastoma (GBM) is the most common primary brain tumor in adults, with a median survival of 15–18 months. GBM cells, like all tumors, exhibit a metabolic shift known as the Warburg effect, favoring glycolysis even under normoxic conditions. GLUT1 is a primary glucose transporter in GBM cells and has been found to be overexpressed in these cells. The acidic microenvironment created by glycolysis facilitates immune evasion, therapy resistance, and tumor growth. Overexpression of GLUT1 is driven by hypoxia-inducible factor-1α (HIF-1α), c-Myc, and other pathways which have been correlated with tumor aggressiveness as well as poor prognosis Recent studies have highlighted the therapeutic potential of targeting GLUT1 in GBM. Preclinical research shows that GLUT1 inhibitors, such as WZB117 and BAY-876, effectively impair tumor metabolism, reduce cell viability, and improve survival in vitro and in animal models. GLUT1 expression also serves as a prognostic marker, with elevated levels linked to poor outcomes. This review highlights the importance of GLUT1 in GBM biology as a potential therapeutic target and biomarker.

## 1. Introduction

Glioblastoma (GBM) is among the most aggressive primary brain tumors in adults, with a 1-year overall survival rate of 53.7% in IDH wildtype and 76.3% in IDH mutant [1]. Despite extensive research into therapeutics capable of targeting GBM cells, these tumors rapidly develop resistance to various treatments, rendering them ineffective [2,3]. GBM cells, like many cancer cells, can proliferate without external stimuli and are highly metabolically active. They can switch from oxidative phosphorylation to anaerobic glycolysis to satisfy their metabolic needs—a shift towards pyruvate utilization historically known as the Warburg effect, a phenomenon observed in cancer cells [4]. This adaptation allows cancer cells to thrive independent of hypoxia or normoxic environments, which is a limiting factor in the metabolism of normal cells.

Over the last century, the discovery and study of proteins involved in this metabolic shift, such as glucose transporters (GLUTs), lactate dehydrogenases (LDHs), hexokinases (HKs), phosphofructokinases (PFKs), pyruvate kinase M2 (PKM2), and monocarboxylate transporters (MCTs) have been pivotal [5,6,7,8,9,10]. Long non-coding RNAs (lncRNAs), microRNAs (miRNAs), and c-Myc have also been identified as direct regulators of the Warburg effect [5,11,12]. This metabolic reprogramming contributes to an acidic tumor microenvironment, promotes resistance to therapies, facilitates gene mutations, supplies nutrients to the biomass, and affects interactions with the immune system [13]. Two key players in the regulation of this metabolic reprogramming are GLUTs and the hypoxia-inducible factors (HIFs).

In GBM, glucose transporters (mainly GLUT1 and GLUT3) regulate glucose uptake into cells. GLUT1 is specifically involved in the internalization of glucose in tumor cells [13]. HIFs are expressed under conditions of reduced oxygen concentration [14]. HIFs, in turn, enhances the expression of GLUT1, enabling cells to remain metabolically active in low-oxygen environments. This increased expression of GLUT1 in GBM cells has been correlated with tumor proliferation and poor patient survival outcomes [15,16]. In this literature review, we explore the role of GLUT1 in the progression of GBM, summarizing previous studies and their findings. We also briefly discuss the challenges and prospects of targeting GLUT1 as a therapeutic strategy.

### 1.1. The Role of GLUT1 in Glucose Transport, Cancer Metabolism, and Tumor Progression

GLUT1, encoded by the *SLC2A1* gene in humans, is a member of the glucose transporter family, which allows the uptake of glucose into cells through facilitated diffusion [17]. This transporter enables efficient glucose utilization under hypoxic conditions. It is ubiquitously expressed and present in normal and cancerous cells, with notable abundance on erythrocytes and barrier cells such as those comprising the blood–brain barrier [17]. In its basal state, GLUT1 predominantly resides in the cytoplasm [18]. Upon specific cellular signals, it translocates to the plasma membrane, thereby permitting the influx of glucose into the cell [18]. This translocation is crucial for maintaining cellular energy homeostasis, particularly under conditions of reduced oxygen tension, where it ensures a continuous supply of glucose for glycolysis and subsequent energy production.

The expression of GLUT1 is an important hallmark of many cancers, such as breast cancer, ovarian cancer, prostate cancer, and GBM [19,20,21,22]. Different transcription factors, such as HIF-1α [23], c-Myc [24,25], K-Ras pathway [26], and PI3K/Akt [27,28] pathways, have been implicated in the upregulation of GLUT1 in hypoxic conditions [29]. Growth factors also play an important role in GLUT1 translocation via the PI3K/AKT pathway, which regulates cell proliferation and growth in many cancers [30].

Cancer cells have the ability to alter their metabolic functions under adverse conditions. This alteration allows for tumor cell proliferation and survival. Studies have shown that cancers that express more GLUT1 are more aggressive and are associated with poorer prognosis [21,31,32,33].

Warburg effect [34] allows tumor cells to efficiently utilize glucose for energy, producing lactate as a byproduct. Lactate is then excreted from the cells through lactic acid transporters [monocarboxylate transporters (MCT1-4)], accumulating lactate in the tumor microenvironment [6,35]. This accumulation of lactate results in an acidic environment, which has several effects. It suppresses local immune responses, thereby protecting the cancer cells from immune system attacks, promoting tumor growth and survival by creating conditions that are unfavorable for normal cells but advantageous for cancer cells [35]. The hypoxic environment and lactate build-up influence cell signaling and transcription processes, including the upregulation of HIF-1α and the activation of nuclear factor kappa-B and PI3K kinase pathways [36,37]. This, in turn, enhances GLUT1 expression and glucose uptake, promotes glycolysis, and further drives lactate production [38].

### 1.2. Hypoxia and the Expression of GLUT1 in Cancer Cells

**Figure 1:**
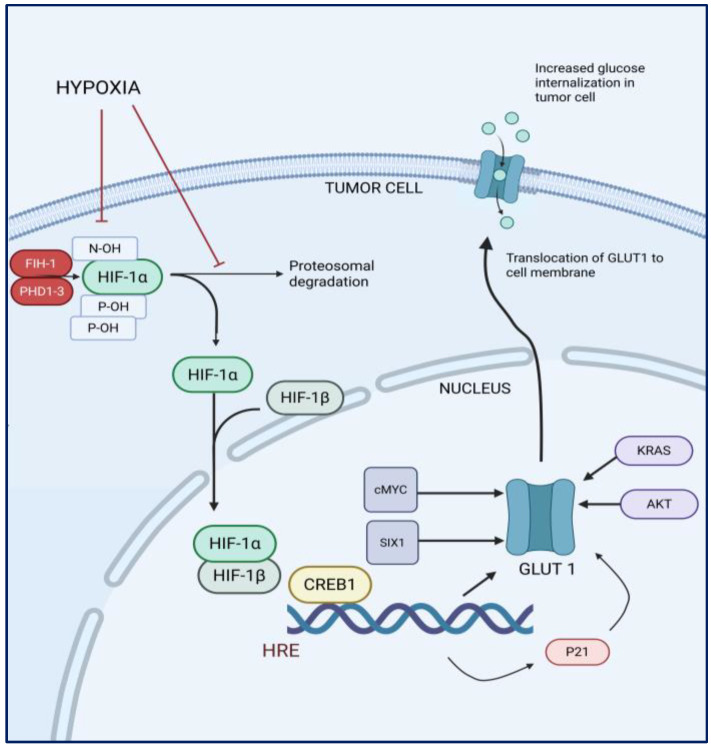
Schematic of hypoxia-induced regulation of glucose uptake in tumor cells.

Under normal oxygen tension, HIF-1α is hydroxylated by prolyl hydroxylase enzymes, which target it for ubiquitination and subsequent proteasomal degradation [39,40]. However, under hypoxic conditions, the activity of prolyl hydroxylases (PHD1-3) and factor inhibiting HIF (FIH-1) is inhibited, leading to the stabilization of HIF-1α. HIF-1α translocates to the nucleus, where it dimerizes with HIF-1β, forming the active HIF-1 transcriptional complex [40]. This HIF-1 complex subsequently binds to specific DNA sequences known as hypoxia-responsive elements (HREs) which contain the core sequence 5′-[A/G]CGTG-3′ in the promoter regions of target genes [38,40]. HIF-1α directly induces the expression of a majority of glycolytic genes by binding to the HREs of the gene promoters (Figure 1). The GLUT1 gene, *SLC2A1*, also contains HREs in its promoter region and when the HIF-1 complex binds to these HREs, it recruits co-activator proteins which possess histone acetyltransferase activity [41]. The binding of HIF-1 and its co-activators to the HREs facilitates the transcription of the GLUT1 gene. The mature mRNA is exported from the nucleus to the cytoplasm, where it is translated into the GLUT1 protein. HIF-1α also regulates the transcription of other molecules, such as vascular endothelial growth factor (VEGF) and carbonic anhydrase IX, which have also been studied in relation to tumor grade and overall survival [42].

Other factors related to hypoxia, including the epidermal growth factor receptor (EGF-R)/KRAS pathway and AKT, also contribute to the upregulation of GLUT1 expression. Transcription factors c-Myc and SIX1 play a crucial role in the regulation of GLUT1 by binding to its upstream promoter regions, thereby enhancing its transcriptional activity. Conversely, the tumor suppressor protein *p53* acts as a regulatory counterbalance by inhibiting the activation of the GLUT1 gene and obstructing the function of the GLUT1 protein [43,44].

### 1.3. The Role of GLUT1 in Glioblastoma

The expression of GLUT1 in GBM cells presents a compelling area of study, given the notable heterogeneity of these tumor cells. In GBM, GLUT1 expression is regulated at both genetic and post-translational levels [45,46]. Various genetic alterations, such as mutations and amplifications, can influence GLUT1 expression, leading to differential glucose uptake among tumor cells [33]. Additionally, post-translational modifications, including phosphorylation and ubiquitination, further modulate the activity and localization of GLUT1 within the cell.

Various mechanisms regulating GLUT1 expression and its pathways have been identified in the context of GBM. In 2022, Jin et al. found that p21 induced by HIF-1α under hypoxic conditions, leading to increased expression of GLUT1 and hence may have an impact on glycolysis-related genes [46]. Another study using the U87 glioma cell line identified GLUT1 as a key target gene regulated by CREB1 (cAMP-responsive element-binding protein 1) that has been implicated in many cancers [45]. It was further observed that both CREB1 and GLUT1 expression levels were significantly elevated in GBM compared to normal brain and WHO grade I, II and III glioma samples, with CREB1 positively influencing the transcriptional activity of GLUT1, thereby enhancing its expression [45].

Zhang et al. and colleagues investigated the mechanism of GLUT1 translocation and identified DHHC9, a palmitoyl transferase, as a key regulator in U87 and T98G glioma cell line [47]. They discovered that DHHC9 catalyzes the palmitoylation of GLUT1 at the cysteine residue Cys207. This post-translational modification is crucial for the translocation of GLUT1 from the intracellular compartments to the plasma membrane, where it facilitates glucose uptake. Inhibition of DHHC9 led to significantly reduced levels of GLUT1 on the cell surface. Consequently, the decreased GLUT1 expression impaired cell survival both in vitro and in vivo in athymic mice intracranially injected with U87, highlighting the importance of DHHC9 in regulating GLUT1 function and cellular metabolism [47].

Several studies have investigated the role of microRNAs (miRNAs) in regulating the expression of GLUT1 [48,49,50]. These miRNAs influence GLUT1 expression by targeting key pathways involved in its transcription and translation. For example, a study by Yin et al. (2020) demonstrated that overexpression of miR-181b in GBM cells significantly inhibited glucose metabolism and cell proliferation [51]. This effect was mediated through the downregulation of Specificity Protein 1 (SP1), a member of the Sp/KLF (Krüppel-like factor/Specificity Protein) family of transcription factors, which are known to regulate gene expression in both physiological and pathological contexts [51]. Abnormal N-glycosylation also plays a significant role in glioma progression, with N-acetylglucosaminyltransferase I (MGAT1) being crucial in converting high-mannose cores into complex or hybrid N-linked oligosaccharides [52]. Elevated *MGAT1* expression has been observed in GBM compared to normal brain tissues [52]. Knockdown of *MGAT1* impaired glioma cell proliferation and migration. Moreover, *MGAT1* facilitated the complex N-glycosylation of GLUT1, increasing GLUT1 protein levels [52]. Furthermore, Cheng et al. demonstrated in an in vitro study using the T98G human GBM cell line that the transcription factor SREBP2 (sterol regulatory element-binding protein-2), known for promoting de novo cholesterol synthesis, also regulated GLUT1 expression. Elevated *SREBP2* expression in T98G cells increased GLUT1 levels, significantly enhancing cell viability and migration [53]. These findings suggest a feedback mechanism that sustains high glucose and cholesterol levels, which are crucial for GBM cell survival.

Together, these complex regulatory mechanisms highlight the tightly controlled expression and function of GLUT1, reflecting its role in cellular metabolism and the adaptive response of GBM cells to their microenvironment.

### 1.4. GLUT1 as a Therapeutic Target in Glioblastoma

**Figure 2:**
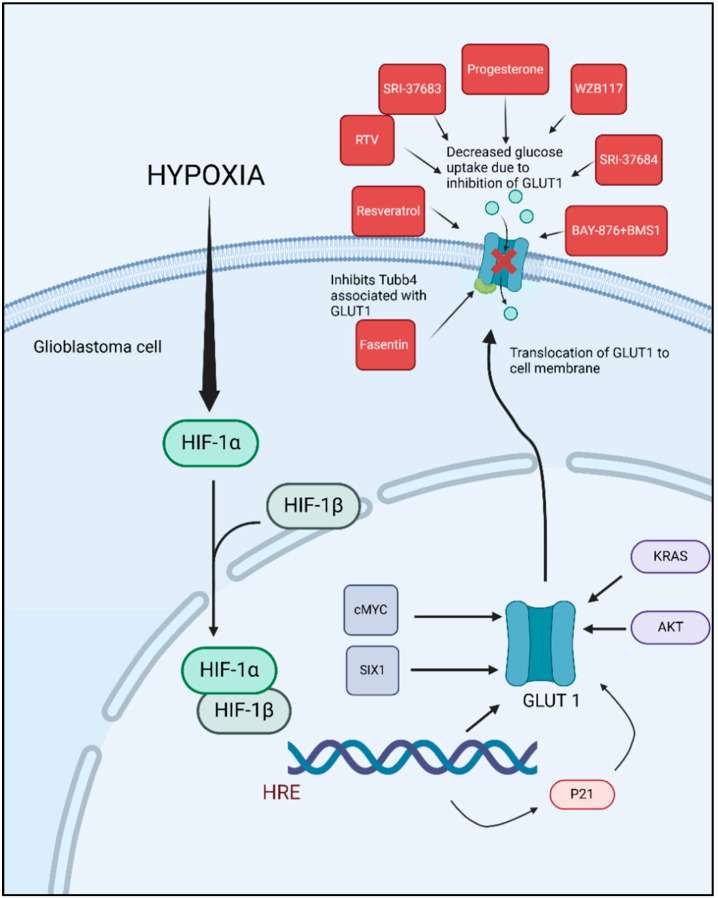
Hypoxia-induced GLUT1 upregulation in glioblastoma and pharmacological inhibition of glucose uptake. GLUT1 inhibitors studied in preclinical GBM models.

Examining potential strategies to target GLUT1 in GBM reveals promising avenues to inhibit tumor growth. Current research has focused on developing and evaluating inhibitors that can specifically target GLUT1, given its critical role in the glycolytic metabolism of GBM and many other cancer cells. These inhibitors aim to disrupt glucose uptake in tumor cells, thereby impeding their energy supply and growth (Figure 2). The crystal structures of human GLUT1 and GLUT3 have elucidated the molecular intricacies of substrate binding and the transport mechanism. These structural insights suggest that inhibitors can be strategically designed to either obstruct substrate binding or disrupt the conformational changes necessary for the transporter’s alternating access mechanism.

The potential of targeting GLUT1 as a therapeutic approach in GBM is promising. Several studies have explored the inhibition of GLUT1, as researchers aimed to disrupt the glucose metabolism of tumor cells, thereby hindering their growth and survival (Table 1). One of these studies demonstrated that targeting GLUT1 and its associated protein TUBB4 with Fasentin significantly reduced cell viability, sphere formation ability, and cell aggressiveness in vitro in patient-derived GSC lines, GSC33 and GSC28 [16]. Fasentin’s inhibition of GLUT1 disrupts glucose uptake, which is critical for energy production and cell survival. Additionally, interfering with TUBB4 affects the structural integrity and function of the cytoskeleton, further impairing the cells’ ability to proliferate and form spheres, a characteristic of aggressive cancer cells [16].

Many studies have explored the effects of chemotherapeutic drugs that act as GLUT1 inhibitors in GBM cell lines. For instance, Azzalin et al. evaluated the effects of Indinavir (IDV) and Ritonavir (RTV) in combination with TMZ and BCNU, in U87GM, Hu-197, GL261 and GBM-P1 (patient derived) cell lines, on the inhibition of the GLUT1/*SLC2A* transporter. They found that RTV, but not IDV, decreased glycolytic activity and cell growth in vitro. Furthermore, mice treated with RTV and BCNU showed improved survival compared to those treated with BCNU alone [54]. Another study conducted both in vitro and in vivo studies with the GL261 mouse glioma cell line, to evaluate the efficacy of BAY-876, a GLUT1 inhibitor. Their research demonstrated that BAY-876 significantly prolonged the survival of mice in an orthotropic GBM model. Furthermore, combining BAY-876 with the PD-1/PD-L1 blocker BMS-1 resulted in an even greater extension of survival, indicating a potential synergistic effect between GLUT1 inhibition and immune checkpoint blockade in the treatment of GBM [56]. In another study, the GLUT 1 inhibitors, SRI-37683 and SRI-37684 effectively decreased the glycolytic capacity, glycolytic reserve capacity, and overall cell survival of PDX GBM cell lines. The findings suggest that targeting GLUT with these compounds can significantly impair the metabolic function and viability of GBM cells [58]. Resveratrol, a natural phenol or polyphenol compound, has shown success in GBM therapy. It exerts its anti-tumor effects through multiple mechanisms, including the regulation of cell cycle progression and proliferation, modulation of autophagy, influence on the oxidative stress system, and activation of apoptosis pathways [70]. Multiple in vitro and in vivo studies have shown decreased tumor cell viability, decreased tumor growth and prolonged survival in the in vivo treatment cohorts [59,60,61,62,65] (Table 1) While studies have not yet demonstrated GLUT1 inhibition by resveratrol in GBM cells specifically, multiple studies in other cell lines like ovarian cancer cell lines [71,72] leukemia cell lines [73] and hypopharyngeal carcinoma [74] have shown that resveratrol can inhibit GLUT1. This suggests that resveratrol may have the potential to affect glycolysis in GBM and could be explored as a therapeutic option.

Currently, resveratrol is the only known GLUT1 inhibitor in the clinical trials phase for cancers such as multiple myeloma, gastrointestinal tumors, follicular lymphoma and breast cancer (Table 2). However, resveratrol is also a known inhibitor of various other pathways, such as STAT3, PI3K, AKT, mTOR, and NF-kB pathways that inhibit cancer progression, which is why it has been used in cancer research [63,66,67,69,71,72]. Over time, researchers discovered that resveretrol also inhibited GLUT1.

In addition, although targeting GLUT1 presents a promising therapeutic strategy, several challenges exist in developing effective drugs for it. The most important is that GLUT1 is ubiquitously expressed in many tissues, including normal cells and vital organs, which increases the risk of off-target effects and toxicity. Ensuring that drugs effectively reach and penetrate tumor cells while sparing healthy tissues poses a significant challenge, especially in the context of GBM due to the blood–brain barrier. In addition, designing drugs that specifically target GLUT1 without affecting other glucose transporters (e.g., GLUT2, GLUT3 or GLUT4) or essential cellular functions can be difficult. The expression of GLUT1 in GBM cells varies significantly, influenced by intra- and inter-tumor heterogeneity and the cells’ proximity to blood vessels. Future research should focus on overcoming these challenges by developing more selective GLUT1 inhibitors, exploring combination therapies that target multiple metabolic pathways, and investigating the mechanisms underlying resistance to GLUT1-targeted treatments. Additionally, advanced imaging techniques and molecular diagnostics can enhance the accuracy of GLUT1 expression assessment, providing more precise and real-time monitoring of therapeutic responses.

### 1.5. Clinical and Prognostic Implications of GLUT1 in GBM Therapy

GLUT1’s role in facilitating glucose uptake makes it a critical target for therapeutic interventions aimed at disrupting the metabolic pathways that GBM cells rely on for survival and growth. Targeting GLUT1 directly or modulating its regulatory pathways can potentially impair tumor metabolism and sensitize GBM cells to other treatments. Radiation is one of the mainstays in the treatment of GBM and irradiation can cause changes in tumor metabolism. GLUT1 has also been implicated in conferring radioresistance and playing a potential role in tumor recurrence [46].

In 2021, Prosniak et al. discovered that tumor cells characterized as GLUT1 positive and Nestin negative cells [GLUT1(+)/NES(−)], typically located far from blood vessels, exhibit natural resistance to conventional chemotherapy and radiation [79]. This resistance was attributed to their low proliferative capacity. A prior study in 2015 by Newman et al. showed that Dichloroacetate (DCA), a PDK inhibitor, modified GBM cell metabolism and caused cells to return to oxidative phosphorylation. Through reversal of this metabolic characteristic of GBM it was sensitized to radiation [80]. Furthermore, another study by Lan et al. showed that a drug, miR-448, negatively affecting glycolysis increased the radiosensitivity of GBM cells in both in vitro and in vivo models [81]. Another study revealed that a decrease in the heat shock protein 90 beta family member 1 leads to reduced GLUT1 localization on the plasma membrane and an ultimate decline in glycolytic activity, resulting in desensitization to radiotherapy [82].

Assessing GLUT1 expression levels can help evaluate the efficacy of such targeted therapies. For instance, a decrease in GLUT1 expression following treatment could indicate a successful disruption of tumor metabolism and a positive therapeutic response [83]. Conversely, as observed in colorectal and acute myeloid leukemia, persistent or increased GLUT1 expression may signal resistance to therapy, prompting the need for alternative or combination treatments [84,85].

Komaki et al. and colleagues observed an increased expression of GLUT1 in pseudo-palisaded and perivascular tumor cells and found its expression to be an independent predictor of worse prognosis in GBM patients [33]. Another study by Guda et al. obtained the mRNA expression data for GLUT1 from the TCGA (The Cancer Genome Atlas) data portal for their patient cohort and studied the correlation between GLUT1 expression and tumor aggressiveness and patient prognosis [16]. By using a data mining approach, they found that the mesenchymal subtype of GBM cells demonstrated the highest mRNA levels of GLUT1 in comparison to proneural, classical, and neural subtypes [16]. They further found that high-grade gliomas expressed more GLUT1 than low-grade gliomas, and patients who expressed higher GLUT1 levels had worse survivorship [16].

These studies highlight the prognostic implications of GLUT1, revealing its potential to predict disease outcomes and progression. Additionally, they highlight the importance of GLUT1 as a valuable biomarker in GBM diagnostics and therapy, emphasizing its role in identifying tumor characteristics, guiding treatment decisions, and monitoring therapeutic responses. The consistent association of elevated GLUT1 expression with aggressive tumor behavior and poor patient prognosis further reinforces its significance in GBM.

## 2. Conclusions and Future Directions

Glucose metabolism plays a crucial role in energy production, with glycolysis serving as the primary metabolic pathway for GBM cells. GLUT1 plays a critical role in tumor maintenance and progression, contributing to aggressiveness, radiation resistance, and recurrence, making it a promising therapeutic target. Preclinical trials in GBM cell lines have demonstrated encouraging results, showing that inhibiting GLUT1 can impair tumor growth and enhance treatment efficacy.

Most studies rely on commercially available GBM cell lines, which lack the heterogeneity seen in PDX. Future research should prioritize PDX models to improve clinical relevance. Given the heterogeneity in GBM tumor cells, focused ultrasound presents an innovative strategy to temporarily disrupt the BBB, enhancing targeted drug delivery of GLUT1 inhibitors. Beyond treatment, GLUT1’s prognostic value should be further validated and studied. Integrating it into a biomarker panel alongside other molecular indicators could refine glioma diagnosis and treatment stratification. A multi-pronged approach combining selective inhibition, advanced drug delivery, and biomarker integration is essential to the utility of GLUT1 as a therapeutic target in GBM.

However, to validate its therapeutic potential for GBM, it is essential to develop targeted drugs that selectively affect the tumor cells with limited off-target effects [86]. Targeting GLUT1 presents challenges due to systemic toxicity and BBB limitations, and there is a need for alternative strategies to target GLUT1 [87]. Given that GLUT1 is a primary glucose transporter at the blood–brain barrier, future studies should also consider the broader impact of GLUT1 inhibition on normal brain metabolism. Evaluating these systemic effects may provide additional insight into the complex role of GLUT1 in glioblastoma progression and therapeutic response.

A potential approach is by using alternative targets like key regulators of GLUT1 translocation and expression, such as chaperones or membrane trafficking facilitators, for more selective inhibition. For instance, inhibitors of MGAT1 and DHHC9 can indirectly target the expression of GLUT1. In 2022, GAL, a long non-coding RNA (lncRNA) regulating GLUT1 expression, was identified as a negative regulator of GLUT1. Given the tissue-specific expression patterns of lncRNAs demonstrated in the study, targeting GAL in GBM may offer a selective strategy to suppress GLUT1 expression in tumor cells. Additionally, miR-181b, a microRNA shown to downregulate GLUT1, may present a promising tool to suppress GLUT1 function post-transcriptionally. Research into findings tumor-specific untranslated regions (UTRs) that are GLUT1/*SLC2A1*-specific can allow for targeted tumor GLUT1 suppression without affecting other normal cells. Developing mutation-activated GLUT1 inhibitors that selectively target GBM-specific mutations such as *EGFRvIII, IDH1, and PTEN* loss, reducing toxicity in normal tissues. Another strategy involves combination therapy, integrating GLUT1 inhibitors with mTOR inhibitors, autophagy blockers, anti-angiogenic agents, or immunotherapies to prevent metabolic adaptation. Immunotherapy in particular could be a promising avenue. A recent report by Jackson et al. highlighted the expression of GLUT1 in myeloid derived suppressor cells (MDSCs) especially in the early-stage MDSCs, an important immunosuppressive population within the GBM microenvironment [88]. GLUT1 can potentially be used to target this population of cells with checkpoint inhibitors to enhance the function of T-cells. Given the central role of metabolic pathways in GBM progression, targeting alternative Warburg effect regulators like *HK2, LDHA, or MCT1*, which are more tumor-specific, may offer greater therapeutic benefits. Future research should prioritize GBM-specific inhibitors, metabolic alternatives, combination therapies, and optimized drug delivery systems to enhance efficacy while minimizing systemic side effects.

## Figures and Tables

**Table 1:** Summary of in vitro and in vivo studies investigating drugs targeting GLUT1 in glioblastoma.

Study, Year	Type of Study	Cell Line	Animal Model	Drug	Mechanism of Action	Dose	Results
Guda et al., 2019 [16]	In vitro	GSC33 and GSC28	N/A	Fasentin	TUBB4 inhibitor	Fasentin at 25 µM and 50 µM dose	Decreased sphere formation over 15 days of treatment
Azzalin et al., 2017 [54]	In vitro and In vivo	U87MG, Hu197 (stable human GBM cell line) and GBM-P1 (primary human GBM cell culture) And GL261 (mouse GBM cell line)	C57BlC inoculated with GL261 GBM cells orthotopic GBM mouse model	Indinavir (IDV) and Ritonavir (RTV) in combination with TMZ and BCNU	Inhibition of GLUT1/*SLC2A* transporter	45 µM and 55 µM of RTV in vitro and BCNU alone (2.5 mg/kg), a combination of RTV (100 mg/kg) and BCNU (1.5 mg/kg), or a combination of RTV (100 mg/kg) and BCNU (2.5 mg/kg) in vivo	Median survival was approximately 18 days for the BCNU-only group and about 37 days for the BCNU 1 + RTV group. RTV but not IDV decreased glycolytic activity and cell growth in vitro. Mice treated with RTV + BCNU have improved survival than BCNU alone, also enhanced
Atif et al., 2019 [55]	In vivo	U118MG (Human GBM cell line), U87dEGFR (modified human cell line) and U87MG-luc	Adult male athymic nude mice inoculated with U87MG-luc orthotopic GBM mouse model	Progesterone	Inhibition of GLUT1, GAPDH and cytoplasmic FoxO1 activity	10–80 µM in vitro and low dose (8 mg/kg) and high dose (100 mg/kg) in vivo	Progesterone treatment significantly enhanced the survival of tumor-bearing mice by 43% compared to vehicle-treated mice without toxicity
Tianliang et al., 2024 [56]	In vitro and In vivo	Mouse GBM cells of GL261, Human GBM cell lines of U87 and U251, Mouse macrophage cell lines of RAW264.7	C57BL/6J mice subcutaneously injected with GL261 cells And C57BL/6J mice inoculated with GL261 or GL261-luc orthotopic GBM mouse model	BAY-876 and PD-1/PD-l1 blocker BMS-1	Inhibitor of GLUT1	1, 2, 4 ng mL^−1^ of BAY-876 in vitro and 2 nm of BAY-876 in vivo	The median survival of tumor-bearing mice treated with BAY-876 increased to 36.5 days compared to 28.5 days in the control group. Treatment with BMS-1 further prolonged survival to 45 days (endpoint)
Shibuya et al., 2015 [57]	In vitro and In vivo	GS-Y03 GBM stem cell and other cancer stem cells (PANC-1 CSLC, and A2780)	male BALB/cAJcl-nu/nu mice subcutaneously implanted with GS-Y03 cell line and other cancer stem cells	WZB117	GLUT1 inhibitor	WZB117 4 mg/mL in DMSO was diluted in PBS to prepare 200 μL for each injection in vivo	In vivo, the tumor size between control and WZB117-treated tumor-bearing mice was statistically significant, with the treated group having a smaller tumor volume.
Landis et al., 2018 [58]	In vitro	GBM (PDX) D456, 1016, JX12, and JX14	N/A	SRI-37683 and SRI-37684	GLUT 1 inhibitor	50 µM SRI-37683 and SRI-37684	There was a 40–50% inhibition of glucose uptake in all tested cell lines
Leone S. et al., 2008 [59]	In vitro	U-87MG	N/A	Resveratrol	GLUT1, STAT3 inhibitor	20 µM of resveratrol and 5 Gy; 180 KV X-rays	20 µM of resveratrol and 5 Gy; 180 KV X-rays
Yand YP et al., 2012 [60]	In vitro and In vivo	CD133 and CD133^+^ cells from a patient	SCID mice (BALB/c strain) orthotopic GBM mouse model	Resveratrol	GLUT1, STAT3 inhibitor	RV 100 µM 2, 4, 6, 8 and 10 Gy	Suppression of STAT3 and induction of apoptosis of tumor cells. Treatment group median survival was 10 weeks vs. 4 weeks in the control group
Wang L et al., 2015 [61]	In vitro and in vivo	Human Glioma SU-2 patient derived	male nude (BALB/c) mice	Resveratrol	GLUT1, STAT3 inhibitor	RV 75 µmol/L (for in vitro) and 150 mg/kg/day (for in vivo) *ip,* and 2, 4 and 6 Gy; 6 MV X-rays	Increased radiosensitivity of cells, prevention of renewal and stemness, increased apoptosis, inhibition of DNA repair. Decreased relative tumor volume in nude mice in treated group
Khoei S et al., 2016 [62]	In vitro	U87MG GBM cell line	N/A	Resveratrol	GLUT1, STAT3 inhibitor	RV 20 µM and 2 Gy; 1.25 MeV	Decreased colony number, increased DNA damage and increased radiosensitivity
Ozturk Y et al., 2019 [63]	In vitro	DBTRG GBM cell line	N/A	Resveratrol	GLUT1, STAT3 inhibitor	RV 50 µM and 50 µM of paclitaxal	Increased mitochondrial ROS levels and activation of TRMP2 channel, increased caspase activity and Ca+ influx through TRMP2 channel
Huang H. et al., 2012 [64]	In vitro	T98G GBM cell line	N/A	Resveratrol	GLUT1, STAT3 inhibitor	RV 100 µM and TMZ 100 µM	Increased chemosensitivity, increased apoptosis, decreased intracellular translocation of NF-kB, and repression of MGMT expression
Li H et al., 2016 [65]	In vitro and in vivo	GBM initiating cells (GICs) from 2 patients	female NOD/SCID mice inoculated by GICs	Resveratrol	GLUT1, STAT3 inhibitor	RV 20 and 40 µM and TMZ; 200 and 400 µM (for in vitro); RV 12.5 mg/kg/day *ip* and TMZ 68 mg/kg/day (in vivo); oral	Induction of apoptosis and activation of p53/pATR/pATM/DSBs pathways, inhibition of self renewal and decreased cell stemness, inactivation of STAT3 and decreased tumor volume
Yang HC et al., 2019 [66]	In vitro and in vivo	U251, T98G, U138, A172, LN229, and normal human astrocytes	N/A	Resveratrol	GLUT1, STAT3 inhibitor	RV 2, 4, 8, 10, 16 and 32 µM and TMZ; 400 µM (in vitro); RV 10 mg/kg/day; *ip* and TMZ 25 mg/kg//day (in vivo)*; ip*	Decreased cell viability and proliferation, increased apoptosis, supression of Wnt pathway and repression of MGMT expression
Liu Y et al., 2020 [67]	In vitro	RG-2, LN-18, LN-428	NA	Resveratrol	GLUT1, STAT3 inhibitor	RV 25, 50, 75 and 100 µM and TMZ 250, 500, 750 and 1000 µM	Inhibition of cells growth repression of MGMT expression, decreased STAT3 decreased Bcl2
Yuan Y et al., 2012 [68]	In vitro and in vivo	SHG44 GBM cell line	Female BALB/cA nude mice inoculated with SHG44 orthotropic glioma model	Resveratrol	GLUT1, STAT3 inhibitor	RV 10 µM and TMZ 100 µM (in vitro); RV 40 mg/kg/day *ip* and TMZ 68 mg/kg/day (in vivo) oral	Cell cycle arrest, downregulation of MMP9 expression, inhibition of cell migration, increased mitochondrial ROS, downregulation og Bcl2, inhibition of mTOR and increased expression of GFAP. Decreased tumor volume
Lin CJ et al., 2012 [69]	In vitro and in vivo	U87 MG and GBM8401 GBM cell lines	female nude mice BALB/c nu/nu subcutaneously injected with U87MG cells	Resveratrol	GLUT1, STAT3 inhibitor	RV 10 µM and TMZ; 100–400 µM (in vitro); RV 12.5 mg/kg/day *ip* and TMZ 10 mg/kg/day (in vivo) *ip*	Increased cell death, increased apoptosis, chemosensitivity. Decreased tumor volume. Decreased ERK activity and LC3-II protein levels and increased cleavage of PARP

Abbreviations: Akt, protein kinase B; BCNU, 1,3-bis(2-chloro-ethyl)-1-nitrosourea (carmustine); BAY-876, selective GLUT1 inhibitor BAY-876; BMS-1, PD-1/PD-L1 checkpoint blocker BMS-1; dEGFR, delta epidermal growth-factor receptor (EGFRvIII); DMSO, dimethyl-sulfoxide; FOXO1, forkhead box O1; GAPDH, glyceraldehyde-3-phosphate dehydrogenase; GBM, glioblastoma multiforme; GIC, glioma-initiating cell; GL261, murine glioma cell line GL261; GLUT1, glucose transporter 1; GSC, glioblastoma stem cell; Gy, gray (radiation dose); IDV, indinavir; ip, intraperitoneal; MGMT, O6-methyl-guanine-DNA methyl-transferase; mTOR, mechanistic target of rapamycin; PANC-1 CSLC, pancreatic cancer stem-like cell PANC-1; PD-1, programmed cell-death protein 1; PD-L1, programmed death-ligand 1; PDX, patient-derived xenograft; PI3K, phosphatidyl-inositol-3-kinase; RV, resveratrol; ROS, reactive oxygen species; RTV, ritonavir; SCID, severe combined immunodeficiency; STAT3, signal transducer and activator of transcription 3; TMZ, temozolomide; TRPM2, transient receptor potential melastatin 2; TUBB4, tubulin-β4; WZB117, small-molecule GLUT1 inhibitor WZB117.

**Table 2:** Clinical trials investigating the use of the GLUT1 inhibitor resveratrol in different cancers.

Trial ID	Year	Cancer Type	Resveretrol Dose	Phase	Country	Results
NCT01476592	2011	Low grade gastrointestinal tumors	2.5 g/2 times a day for three cycles, oral	Not applicable	USA	Study completed in 2017 but results are unavailable
NCT00920803 [75]	2008	Colorectal cancer and hepatic metastasis	SRT501 5.0 g daily for 14 days, oral	Phase 1	UK	Hepatic tissue was found to contain resveratrol
NCT00256334 [76]	2005	Colon cancer	Four dose cohorts—plant derived resveratrol tablets 80 mg/day or 20 mg/day or Freeze dried grape powder (GP) (containing reveretrol) at 120 mg/day or 80 mg/day, oral	Phase 1	USA	GP was successful in tarteting and inhibiting *Wnt* target gene,
NCT00920556 [77]	2009	Multiple Myeloma	SRT501 5 g/day for 20 days in a 21 day cycle for upto 12 cycles, oral	Phase 2	UK	Unacceptable safety profile with minimal efficacy in recurrent multiple myeloma.
NCT00433576 [78]	2006	Colorectal cancer	Oral resveretrol for 9 days	Phase 1	USA	Study completed in 2009 but results are unavailable. The objective of the study was to determine oral dose and colon mucosal levels and correlate levels of COX-2 and M1G in colorectal cancer tissue and WBCs

## Data Availability

No new data were created.
